# The microbial communities and natural fermentation quality of ensiling oat (*Avena sativa* L.) harvest from different elevations on the Qinghai-Tibet Plateau

**DOI:** 10.3389/fmicb.2022.1108890

**Published:** 2023-01-11

**Authors:** Yuhong Bao, Zhaxi Yangzong, Zhenjie Yuan, Ruizhi Shi, Ke Feng, Pengcheng Xin, Tianzeng Song

**Affiliations:** ^1^Institute of Grass Science, TAR Academy of Agricultural and Animal Husbandry Sciences, Lhasa, China; ^2^State Key Laboratory of Germplasm Resources and Genetic Improvement of Tibetan Barley and Yak, Lhasa, China; ^3^Institute of Animal Science, TAR Academy of Agricultural and Animal Husbandry Sciences, Lhasa, China; ^4^Animal Disease Prevention and Control Center of Lhasa, Lhasa, China; ^5^Maiji District Animal Husbandry Technology Promotion Station, Tianshui, China

**Keywords:** elevational gradients, fermentation quality, microbial community, oat, Tibetan Plateau

## Abstract

**Introduction:**

Ensiling whole-crop oat (*Avena sativa* L.) has attracted a growing interest in the Qinghai-Tibet Plateau. The study aimed to investigate the microbial community and chemical composition of fresh and ensiling oat harvested from six different elevations of the Qinghai-Tibet Plateau.

**Method:**

The oat (*A. sativa* L. cv. Qingyin No*.* 1) was planted in six different sites across Qinghai-Tibet Plateau (BM, Bomi County; BY, Bayi County; DZ, Dazi County; BR, Biru County; SC, Suo County; SN, Seni County), where the elevations were in the range of 2,800–4,500 m above sea level (a. s. l.). Oat was harvested at the milk stage and ensiled for 90 days.

**Results:**

The highest crude protein (CP) and lowest water-soluble carbohydrate (WSC) were observed in fresh oat of SN and BM, respectively, however, no distinct gradient trend in WSC and CP concentrations along the elevation gradient. The lowest LAB counts in fresh oat from the highest elevational regions of SN. After 90 days of ensiling, the pH in all oat silages was lower than 4.2, and silages from SC and SN showed a lower pH and butyric acid concentration, and higher lactic acid (LA) concentration than silages of other regions. The oat silage from BR showed the lowest LA concentration and the highest pH. The bimodal distributions of fungal and bacterial richness in fresh oat along the elevation gradient were observed, while the elevation gradients did not affect the fungal Shannon index in fresh oat. *Dioszegia*, *Cladosporium*, and *Vishniacozyma* were the prevalent fungal genus in fresh oat, while *Wickerhamomyces*, *Candida*, and *Saccharomyces* dominated the fungal communities of silages. *Wickerhamomyces* and *Candida* were the dominant genera in oat silages from BM and SC, respectively. *Erwinia*, *Paenibacillus*, *Pseudomonas*, *Leuconostoc*, and *Exiguobacterium* dominated the bacterial community of fresh oat, while *Lactobacillus* and *Kosakonia* were the dominant bacterial genus in oat silages. *Pantoea* was the most dominant bacterial genus in fresh oat from low-elevational regions (BM, BY, and DZ). Oat from SN exhibited the best fermentation quality although fresh oat of SN hosted the lowest LAB counts, indicating that high-efficient LAB might be present in fresh oat sampled from high altitudes.

## Introduction

The Qinghai-Tibet Plateau is the largest and highest plateau with a unique and fragile ecosystem because of high ultraviolet radiation, low precipitation, and low temperatures, which restrained crop growth (Wu et al., 2022). Tibetan living on the Qinghai-Tibetan Plateau rely for survival on the yaks (*Bos grunnien*s) since ancient times (Guo et al., 2014), which is a unique bovine well adapted to the harsh alpine environmental conditions and extensive grazing management all year round. However, the dramatic climate change and intensive grazing activities in past decades have caused gradual rangeland degradation, reducing the availability of herbage (Ding et al., 2014). The reallocation and transporting of forage became an effective management practice to prevent grassland degradation and ensure the sustainability of the livestock industry. Cultivation and ensiling forage crops in arable regions of the Qinghai-Tibet Plateau would provide the available forage resources for the spatiotemporal allocation of forages.

Oat (*Avena sativa* L.) is a common forage crop cultivated on the Qinghai-Tibet Plateau because of its strong drought resistance, short growth cycle, high forage yield, good palatability, and high nutritional value. Ensiling whole-crop oat has attracted a growing interest in the Qinghai-Tibet Plateau, and oat silage became an important relief and emergency fodder to alleviate the forage shortage of cattle and sheep during the cold season. Ensiling fermentation is a traditional technique to preserve the original composition of nutrients based on LA fermentation under anaerobic conditions. The harsh natural environment and unique climates might contribute to the difference in the epiphytic microbial community and chemical composition of forages between the Qinghai-Tibet Plateau and other regions worldwide (Ding et al., 2020). Moreover, the large elevation gradient across the Qinghai-Tibet Plateau enlarged the discrepancy in microbial and chemical composition in crops cultivated at different elevations. Previous studies revealed the altitudinal distribution patterns of phyllosphere microbial communities and silage fermentation of the wild grass of *Kobresia pygmaea* and *Elymus nutans* along the elevation gradient on the Qinghai-Tibet Plateau (Ding et al., 2020; Yang X. et al., 2022). However, there are limited studies on the altitudinal distribution patterns of phyllosphere microbial communities and chemical composition in cultivated grass, which might be different from that of wild grass because more human activities were involved in the cultivation during the crop growth.

The study aimed to investigate the microbial community and chemical composition of fresh and ensiling oat harvested from six different elevations of the Qinghai-Tibet Plateau. It was hypothesized that the microbial community and chemical composition were different among elevations, which contributed to the discrepancy in the fermentation quality of oat silage harvested from six regions of the Qinghai-Tibetan Plateau.

## Materials and methods

### Sampling area and silage preparation

The oat was grown at six different sites across arable regions of Qinghai-Tibet Plateau (BM, Bomi County; BY, Bayi County; DZ, Dazi County; BR, Biru County; SC, Suo County; SN, Seni County), where the elevation was in the range of 2,800–4,500 m above sea level (a. s. l.). The geographic location and climate characteristics of these sites are shown in Supplementary Table S1. The oat variety of *Avena sativa* L. cv. Qingyin No. 1 was planted in six sites and underwent *similar* field management measures. Oat was harvested at the milk stage from 20 August to 5 September 2021. Three plots (100 m × 50 m block) were set for each site, and the oat from each plot was chopped in 1–2 cm lengths and packed into polyethylene plastic bags (dimensions 270 mm × 300 mm) followed by vacuum-sealing. The three plots served as replications for each elevation. The bags were taken to Lhasa and stored for 90 days at an ambient temperature (10–24°C). The fresh forages from each plot were sampled for chemical analyses, microbial counting, and DNA extraction.

### Laboratory analyses

The silage bags were opened on d 90 of ensiling, and all silages of each bag were put into an ethanol-sterilized plastic container and mixed thoroughly. Then all silages were divided into 4 sub-samples. The first sub-sample was dried at 65°C in a forced-air oven for 48 h to determine DM contents, the dried samples were ground through a 1-mm sieve by a laboratory knife grinder. The ground samples were preserved for chemical analyses. Total nitrogen (TN) content was analyzed with a Kjeltec 8,400-Analyzer (FOSS Analytical AB, Höganäs, Sweden), and the crude protein (CP) was calculated by TN × 6.25 (Krishnamoorthy et al., 1982). The water-soluble carbohydrate (WSC) content was quantified according to the method of Thomas (1977). The neutral detergent fiber (NDF) and acid detergent fiber (ADF) were quantified according to the Van Soest procedures (Van Soest et al., 1991).

The second sub-sample (20 g wet basis) was extracted with 60 ml distilled water at 4°C for 24 h. The solution was filtered through four layers of medical gauze and Whatman filter paper (Hangzhou Xinhua Co., LTD., China), followed by measuring pH immediately using a pH electrode (Hanna Instruments Italia Srl, Padua, Italy). Then silage extract (5 ml) was centrifuged at 12000× *g* for 10 min at 4°C, and the supernatant was filtered through a 0.45 μm membrane for organic acid and ethanol analyses according to Yang X. et al. (2022). Ammonia N of silage extract was measured by the phenol-hypochlorite method according to Broderick and Kang (1980).

The third sub-sample (10 g) was homogenized with 90 ml of sterile sodium chloride solution (0.85%) for 1 min, followed by filtering through four layers of cheesecloth. Then the filtrate was 10-fold serially diluted for microbial counting, and another solution was used for microbial DNA extraction. The LAB was counted on deMan, Rogosa, and Sharp agar after 48 h of anaerobic incubation at 37°C. The number of yeasts and molds was determined on Potato Dextrose Agar (PDA) medium after 48–72 h of aerobic incubation at 28°C. Enterobacteriaceae were counted on purple-red bile glucose agar after 24 h of incubation at 37°C under aerobic conditions. Aerobic bacteria were counted on the nutrient agar medium (Qingdao Haibo Biotechnology Co., Ltd) after aerobic incubation for 48 h at 37°C. All of the microbial data were transformed to log10 for presentation and statistical analysis.

### Microbial community analyses

Microbial DNA was extracted using the Fast DNA SPIN Kit for Soil (MP Biomedicals, Solon, OH, USA). The bacterial 16S rRNA V3–V4 and fungal ITS regions were amplified with primers 338F-806R and ITS1F-ITS2aR, respectively. After purification, the purified PCR amplicons were paired-end sequenced using the Illumina MiSeq PE300 platform (Illumina Inc., San Diego, CA, USA). All raw reads were checked to discard low-quality sequences (quality scores <20) using FLASH (Version 1.2.11) and QIIME (Version 1.7.0). Operation taxonomic units (OTUs) were clustered based on a 97% sequence similarity cutoff using UPARSE (Version 7.1).^1^ Then the chimeric sequences were identified and removed using UCHIME. Bacterial and fungal compositions were analyzed at genus levels using the Silva and Unite database with a confidence threshold of 70%, respectively. Alpha-diversity estimates (Chao1, and Shannon) and beta-diversity evaluation, based on principal coordinate analysis (PCoA), were performed using the Phyloseq and Vegan packages on R. All DNA sequences have been deposited in the NCBI Short Read Archive database under BioProject PRJNA910079.

### Statistical analysis

The experiment was a completely randomized design, all data from chemical composition, microbial populations, microbial diversity, and fermentation were analyzed by one-way analysis of variance (ANOVA) using the GLM procedure of SAS (version 9.3; SAS Institute Inc., Cary, NC). A polynomial contrast was used to test the linear or quadratic effects of elevation gradient on the parameters measured. The differences between means were assessed by Tukey’s multiple comparisons (*p* < 0.05).

## Results

### Chemical composition and microbial populations in fresh oat before ensiling

The chemical composition and microbial populations of fresh oat harvested from different sites of the Qinghai-Tibet Plateau are listed in Table 1. The DM contents of fresh oat ranged from 232 to 310 g kg^−1^ of fresh weight (FW), and the elevation gradient exhibited a linear effect on DM content (*p* < 0.001). Among all fresh forages, both NDF (*p* = 0.008) and ADF (*p* = 0.003) in fresh oat harvested from BR were the highest. The CP content was affected by elevation gradient with a linear and quadratic effect (*p* < 0.001). The elevation gradient exhibited a quadratic effect on WSC content (*p* < 0.001). The elevation gradient significantly affected the numbers of LAB with linear and quadratic effects (*p* < 0.001). The numbers of yeast and aerobic bacteria were significantly affected by the elevation gradient (*p* < 0.01).

**Table 1:** The chemical composition (g/kg DM basis unless stated otherwise) and microbial populations (log_10_ cfu/g FW) of fresh oat (*Avena sativa* L.).

Item	Altitude^1^						SEM^2^	Value of *p*	Contrast *p*-values	
	BM	BY	DZ	BR	SC	SN			Linear	Quadratic
Dry matter (g kg^−1^ FW)	305^a^	254^b^	249^b^	310^a^	232^b^	241^b^	8.0	<0.01	<0.01	0.902
Neutral detergent fiber	326^b^	489^a^	390^ab^	497^a^	416^ab^	360^ab^	1.8	<0.01	0.826	<0.01
Acid detergent fiber	121^c^	265^a^	197^abc^	248^ab^	217^abc^	163^bc^	1.4	<0.01	0.519	<0.01
Crude protein	85.2^b^	58.7^cd^	77.2^b^	49.5^d^	70.0^bc^	126^a^	0.60	<0.01	<0.01	<0.01
Water-soluble carbohydrate	55.9^d^	174^a^	137^b^	116^bc^	143^ab^	100^c^	9.34	<0.01	0.110	<0.01
Lactic acid bacteria	6.22^a^	6.11^ab^	5.83^b^	6.21^a^	6.03^ab^	4.82^c^	0.121	<0.01	<0.001	<0.01
Molds	3.07	3.33	3.30	3.30	3.30	3.02	0.050	0.293	0.752	0.031
Yeast	5.22^a^	5.04^b^	5.22^a^	5.23^a^	5.21^a^	5.20^a^	0.018	<0.01	0.083	0.742
Enterobacteriaceae	7.01^ab^	6.78^b^	6.69^b^	7.62^a^	6.62^b^	6.90^b^	0.094	<0.01	0.940	0.402
Aerobic bacteria	7.40^bc^	7.61^ab^	7.53^ab^	7.78^a^	7.22^a^	7.68^a^	0.049	<0.01	0.348	0.239

^a − d^ Means within a row with different letters differ (*p* < 0.05).^1^BM, Bomi County; BY, Bayi County; DZ, Dazi County; BR, Biru County; SC, Suo County; SN, Seni County. ^2^SEM, standard error of the mean.

### Fermentation characteristics of oat silages after 90 days of ensiling

The fermentation quality, microbial population, and chemical composition of oat silage are listed in Table 2. There was a quadratic effect (*p* < 0.01) of elevation gradient on the DM and WSC contents. The DZ and SC silages had the lowest DM content, while the BR silage had the highest WSC content among all silages. The ammonia N concentration exhibited a quadratic (*p* < 0.01) response to the elevation gradient with the lowest value in BR silage. The amounts of LAB (*p* = 0.01) and aerobic bacteria exhibited a quadratic response to the elevation gradient (*p* < 0.01).

**Table 2:** The chemical composition, fermentation quality (g/kg DM basis unless stated otherwise), and microbial populations (log _10_ cfu/g FW) of ensiled oat (*Avena sativa* L.).

Item	Altitude^1^						SEM^2^	Value of *p*	Contrast *p*-values	
	BM	BY	DZ	BR	SC	SN			Linear	Quadratic
Dry matter (g kg^−1^ FW)	273^a^	236^bc^	215^d^	254^b^	216^d^	232^cd^	5.21	<0.01	<0.01	<0.01
pH	4.12^a^	4.01^abc^	4.09^ab^	4.15^a^	3.73^bc^	3.71^c^	2.800	<0.01	<0.01	0.047
Lactic acid	89.5^ab^	60.7^bc^	66.3^bc^	31.5^c^	113^a^	73.8^b^	6.61	<0.01	0.484	<0.01
Acetic acid	8.72^b^	10.1^ab^	31.7^a^	16.1^ab^	12.3^ab^	14.3^ab^	2.43	0.044	0.640	0.039
Propionic acid	0.50	0.16	2.57	0.77	0.40	0.15	0.280	0.077	0.554	0.061
Butyric acid	5.58^bcd^	11.6^bc^	26.8^a^	14.5^b^	2.71^cd^	1.53^d^	2.210	<0.01	<0.01	<0.01
Ethanol	10.2^b^	35.6^a^	22.7^ab^	15.3^ab^	18.6^ab^	6.82^b^	2.800	0.014	0.083	0.017
Water-soluble carbohydrate	11.5^b^	8.39^b^	10.6^b^	30.7^a^	11.4^b^	11.6^b^	1.90	<0.01	0.041	<0.01
Ammonia N	13.6^b^	9.39^c^	12.4^b^	7.91^c^	11.2^b^	20.1^a^	0.945	<0.01	<0.01	<0.01
Lactic acid bacteria	7.64^a^	7.3^ab^	6.63^ab^	6.3^b^	7.3^ab^	6.97^ab^	0.140	0.018	0.087	0.010
Molds	<2.30	2.40	<2.30	<2.30	<2.30	<2.30	0.020	0.458	0.397	0.794
Yeast	4.17	3.30	3.47	3.30	3.73	3.30	0.100	0.054	0.080	0.104
Aerobic bacteria	6.27^a^	5.40^b^	5.39^b^	5.30^b^	5.50^b^	5.60^b^	0.09	<0.01	0.013	<0.01

^a − d^Means within a row with different letters differ (*P* < 0.05). ^1^BM, Bomi County; BY, Bayi County; DZ, Dazi County; BR, Biru County; SC, Suo County; SN, Seni County. ^2^SEM, standard error of the mean.

The silage pH decreased in linear (*p* < 0.01) and quadratic (*p* = 0.047) manners along the elevation gradient. The LA concentration was affected by elevation gradient (*p* < 0.01) with the highest LA values in SC. The AA concentration exhibited a quadratic (*p* < 0.01) response to the elevation gradient with the highest AA concentration in DZ. The elevation gradient quadratically affected (*p* < 0.01) the butyric acid concentration. The ethanol concentration exhibited a quadratic (*p* = 0.017) response to the elevation gradient with the highest value in BY.

### Bacterial and fungal community of fresh and ensiling oat

**Figure 1:**
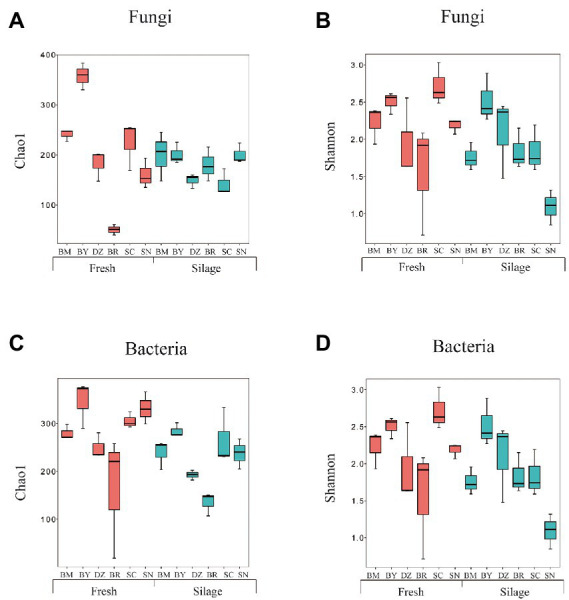
Alpha diversity of fungal **(A,B)** and bacterial **(C,D)** community diversities of fresh and ensiled *oat* along the elevation gradient on the Tibetan Plateau. BM, Bomi County; BY, Bayi County; DZ, Dazi County; BR, Biru County; SC, Suo County; SN, Seni County.

We obtained 2,662,561 and 2,876,965 reads by amplicon sequencing of bacterial 16S rRNA V3-V4 and fungal ITS regions, respectively. The greater coverage (>99%) and plateaued rarefaction curves for all samples indicated that the sequencing depth was adequate for reliable analysis of the bacterial and fungal community. The α-diversity indexes of the bacterial and fungal communities are shown in Figure 1; Supplementary Table S2. The bacterial Shannon index in fresh oat of BR was the lowest among all fresh materials while the bacterial Shannon index in oat silage of SN was the lowest among all silages. The bacterial Chao1 index decreased after 90 days of ensiling. Both fresh and ensiled oat from BR showed the lowest bacterial Chao1 index. The fungal Shannon was similar (*p* > 0.05) among all fresh materials while that in BM and SC silages was lower than in other silages. Fresh oat from BY and BR showed the highest and lowest Chao 1 index among all fresh materials while the elevation gradient did not affect (*p* > 0.05) the fungal Chao 1 index of silages.

**Figure 2:**
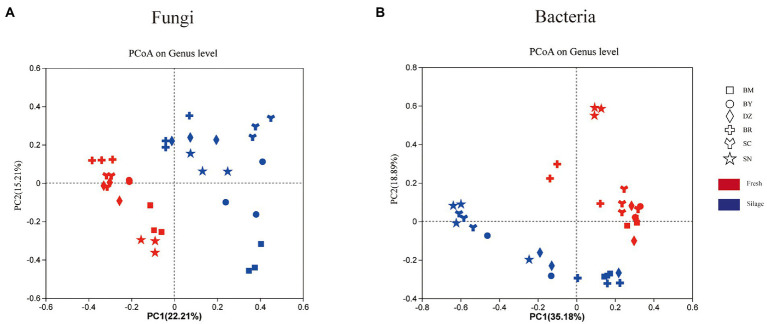
The unweighted Principal coordinate analysis (PCoA) of fungal **(A)** and bacterial **(B)** communities of fresh and ensiled *oat* along the elevation gradient on the Tibetan Plateau. BM, Bomi County; BY, Bayi County; DZ, Dazi County; BR, Biru County; SC, Suo County; SN, Seni County.

The PCoA plot on the unweighted UniFrac showed the separation of bacterial and fungal communities of fresh and ensiling oat (Figure 2). Two principal components accounted for 54.07% of the variation in taxonomic composition among samples, the PC1 and PC2 axis explained 35.18 and 18.89% of the total variation of the bacterial community, respectively (Figure 2B). The fresh oat and 90-days silages were separated in the plot. Among fresh oat, oat harvested from SN was separated from oat of other regions. The silages of SN and SC were clustered together and separated from oat silages from other regions. Two principal components (PC1 and PC2) accounted for 37.42% of the variation in taxonomic composition among samples (22.21 and 15.21%, respectively) for the fungal community (Figure 2A). The fresh forages were clustered in the second and third quadrants, while all silages were dispersed in the first and fourth quadrants.

**Figure 3:**
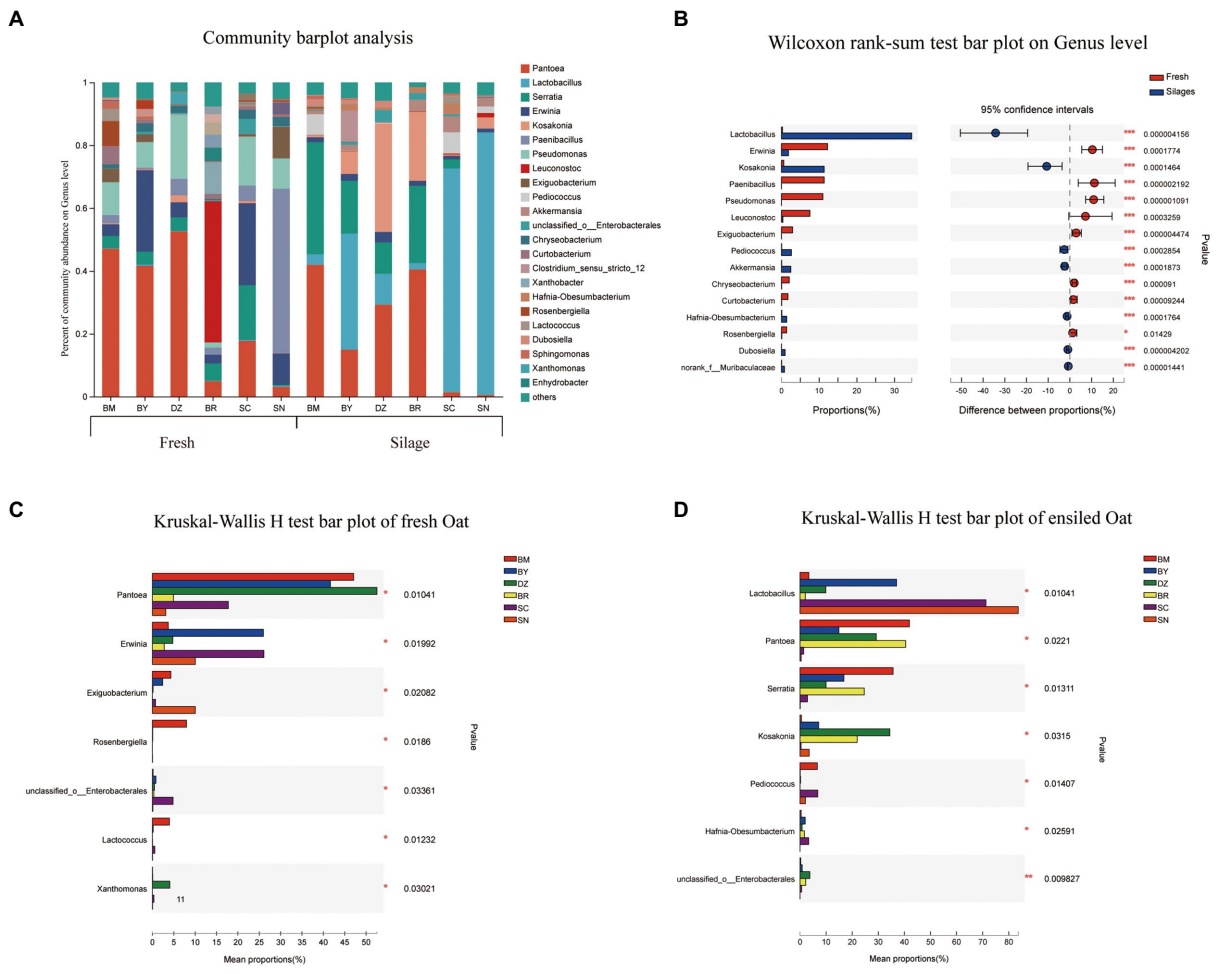
Bacterial community composition of fresh and ensiled oat along the elevation gradient on the Tibetan Plateau. **(A)** The bar plots of bacterial community composition of fresh and ensiled oat at the genus level. **(B)** Extended error bar plot showing the most abundant Bacterial genus that had significant differences between fresh and ensiled oat. Positive differences in mean relative abundance indicate the Bacterial genus overrepresented in fresh oat, while negative differences indicate greater abundance in oat silages. **(C)** Relative abundances of bacterial genera showed significant differences among fresh oat. A one-way ANOVA was used to evaluate the significance of differences between the indicated groups. *p < 0.05,**p < 0.01,***p < 0.001. **(D)** Relative abundances of bacterial genera showed significant differences among oat silages. A one-way ANOVA was used to evaluate the significance of differences between the indicated groups. *p < 0.05,**p < 0.01,***p < 0.001.

The bacterial community composition in fresh and ensiled oat at the genus level are shown in Figure 3. *Erwinia*, *Paenibacillus*, *Pseudomonas*, *Leuconostoc*, and *Exiguobacterium* dominated the bacterial community of fresh oat, while *Lactobacillus* and *Kosakonia* were the dominant bacterial genus in oat silages (Figure 3B). *Pantoea* was the most dominant bacterial genus in fresh oat harvested from low elevational regions (BM, BY, and DZ), and its relative abundances (RA) decreased to 4.9, 17.7, and 3.1% in BR, SC, and SN, respectively. After 90 days of ensiling, the RA of *Pantoea* marked declined except for the oat silage of BR. The RA of *Lactobacillus* in fresh oat was lower than 5%, it markedly increased and became the most dominant genus in oat silages of SC (71.2%) and SN (83.6%) after 90 days of ensiling. *Serratia* was the prevalent bacterial genus in fresh oat, and its RA significantly increased in oat silages of low-elevational regions (BM, BY, DZ, and BR) after 90 days of ensiling. *Erwinia* was present in all fresh oat, and its RA decreased after 90 days of ensiling. *Paenibacillus*, *Pseudomonas*, and *Exiguobacterium* were detectable in all fresh oat and disappeared in all silages. *Leuconostoc* was the most dominant genus in fresh oat from BR, while it was undetectable in fresh oat from other elevations.

**Figure 4:**
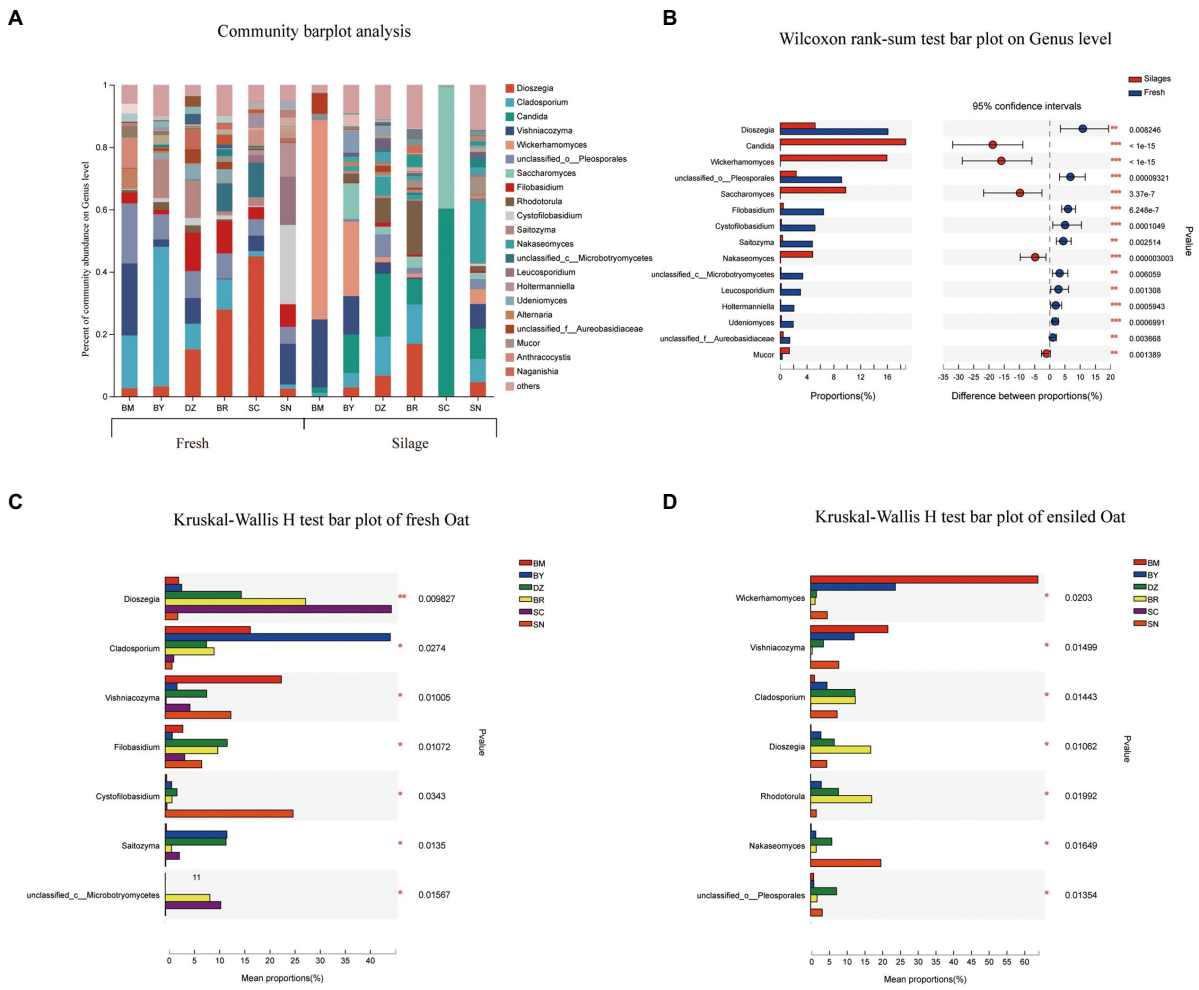
Fungal community composition of fresh and ensiled oat along the elevation gradient on the Tibetan Plateau. **(A)** The bar plots of fungal community composition of fresh and ensiled oat at the genus level. **(B)** Extended error bar plot showing the most abundant Fungal genus that had significant differences between fresh and ensiled oat. Positive differences in mean relative abundance indicate the Fungal genus overrepresented in fresh oat, while negative differences indicate greater abundance in oat silages. **(C)** Relative abundances of fungal genera showed significant differences among fresh oat. A one-way ANOVA was used to evaluate the significance of differences between the indicated groups. *p < 0.05,**p < 0.01,***p < 0.001. **(D)** Relative abundances of fungal genera showed significant differences among oat silages. A one-way ANOVA was used to evaluate the significance of differences between the indicated groups. *p < 0.05,**p < 0.01,***p < 0.001.

The fungal community composition at the genus level in fresh and ensiled oat are shown in Figure 4. *Dioszegia*, *Cladosporium*, and *Vishniacozyma* were the prevalent fungal genus in fresh oat with different RA among elevation gradients. The RA of *Dioszegia* was increased from 2.6% in fresh oat of BM to 44.9% in fresh oat of SC. *Cladosporium* was the most dominant fungal genus in BY and decreased along the elevation gradient. *Vishniacozyma* was the most dominant fungal genus in BM, accounting for RA of 23.1%. After 90 days of ensiling, all 3 of *Dioszegia*, *Cladosporium*, and *Vishniacozyma* were still detectable in silages, however, the dominant role was replaced by other fungal genera. *Wickerhamomyces*, *Candida*, and *Saccharomyces* dominated the fungal communities of silages although they present in very low abundance in fresh oat (Figure 4B). *Wickerhamomyces* and *Candida* were the dominant genera in ensiling oat from BM and SC, respectively.

## Discussion

### Chemical characteristics of fresh oat along the elevation gradient

The elevation is a complicated, indirect gradient along which several environmental variables shift, resulting in a basic diversity gradient trend in plant biogeography (Afzal et al., 2021). The low temperature at high elevational regions restrains plant respiration and results in the accumulation of carbohydrates, proteins, and ether extract in the cell protoplasm, which will be beneficial to decrease its freezing point and enhance its adaptive resistance to cold (Li et al., 2014). Plants growing at higher elevations require relatively more energy to respond to serious environmental stress (Marini et al., 2009). Ding et al. (2020) observed an increase in WSC and CP in fresh *E. nutans* along the increasing elevation. The highest CP and lowest WSC were observed in fresh oat of SN and BM, respectively, however, we did not find a distinct gradient trend in WSC and CP concentrations along the elevation gradient. In the present study, oat is a cultivated forage crop for livestock in the Qinghai-Tibetan plateau, its chemical compositions are not only affected by climate induced by elevation gradients but also influenced by the human activities. Fu et al. (2022) studied the effects of climate change and human activities on forage nutritional quality, they found that the effects of climate change and human activities on nutritional quality of forage were indistinguishable across the whole Tibet, however, human activities altered the sensitivities of forage nutritional quality to climate change.

Sufficient WSC and LAB are the crucial factors for silage fermentation, the WSC in all fresh oat except for BM was above 100 g/kg, which is higher than the recommended level of WSC for well silage fermentation (Woolford, 1984). The LAB counts in fresh oat were higher than 4.8 log_10_ cfu/g FW, which is close to the recommended counts of LAB (10^5^ cfu*/*g FM) for well-fermented silages (You et al., 2021).

The lowest LAB counts in fresh oat from the highest elevational regions of SN might be related to the harsh environment because microbes must overcome the stress induced by the extreme climate of high-altitude regions. Some microorganisms still could adapt to these extreme conditions including low temperatures, high levels of solar radiation, periodic freeze–thaw cycles, and nutrient limitations. Liu et al. (2022) reported that nearly 1,000 microbe species were discovered in ‘extreme’ Tibetan glaciers, and 82% of the genomes were novel species.

### The fermentation characteristics of oat silages after 90 days of ensiling

After 90 days of ensiling, the pH in all oat silages was lower than 4.2, indicating all oat silage exhibited well fermentation quality. Of six oat silages, silages from SC and SN showed the lowest pH and butyric acid concentration, and higher LA concentration. Oat from SN exhibited the best fermentation quality although the fresh oat of SN hosted the lowest LAB counts, indicating that high-efficient LAB might be present in fresh oat sampled from high altitudes. Ding et al. (2020) also observed a higher LA concentration in *E. nutans* silages sampled from Naqu (altitude of 4,752 m) than in other low altitudes (<4,228 m), and they attributed the high LA concentration to the greater LA-fermentation efficiency of LAB on this region. The oat silage from BR showed the lowest LA concentration and the highest pH, which were in line with its highest residual WSC concentration after 90 days of ensiling, indicating the complex epiphytic microbial composition might retard the LA fermentation.

In the present study, butyric acid was observed in all silages, indicating all oat silages had undergone a clostridial fermentation. The production of butyric acid usually results in high losses of dry matter and digestible energy (Vissers et al., 2007). High butyric acid sometimes could induce ketosis in lactating cows. Silage with a high butyric acid is also less palatable and can also promote the onset of ketosis in lactating cows. The oat silage from DZ showed the highest butyric acid concentrations, which was related to its lowest DM content. Buxton and Muck (2003) reported that moist silage (>70% moisture) usually is associated with poor fermentation dominated by undesirable butyric acid-forming bacteria. The highest ethanol content in oat silage of BY might be related to the high WSC in fresh oat. de Oliveira et al. (2016) reported that in some cases, the higher WSC content of forage could cause acid silage and increase ethanol contents due to yeast activity, however, excess ethanol negatively affected the silage quality because it resulted in a decrease in DM intake.

### The altitudinal distribution of microbial community in fresh and ensiled oat

The elevational patterns for plant and animal diversity generally follow a certain pattern, however, microbes do not follow similar and clear elevational diversity patterns with plants and animals (Fierer et al., 2011). The inconsistency of phyllosphere microbial diversity patterns across elevations could be attributed to changing environmental factors induced by elevational variables (Yang N. et al., 2022). Yang X. et al. (2022) found that the phyllosphere bacterial and fungal richness showed a unimodal distribution, phyllosphere fungal diversity decreased while the phyllosphere bacterial diversity increased along the elevational gradient. We observed the bimodal distribution of fungal and bacterial richness in fresh oat along the elevation gradient (2852–4447 m a.s.l). Vacher et al. (2016) demonstrated that the higher dispersal ability of bacteria and higher sensitivity of fungi to temperature variations might account for the bigger response of phyllosphere fungal communities to elevation gradient than bacterial communities. In the present study, the elevation gradients did not affect the fungal Shannon index in fresh oat (*p* > 0.05), which was consistent with the reports by Yang X. et al. (2022), who also detected similar soil fungal Shannon index across elevation gradients.

In the study, *Pantoea* dominated (>41%) the fresh oat of lower elevations (BM, BY, and DZ). This is in line with the reports of Ding et al. (2020), who reported that *Pseudomonas* and *Pantoea* were the dominant bacterial genera in fresh E. *nutans* of Tianzhu (2,965 a.s.l.). Cheng et al. (2022) also found that *Pantoea* was the main microorganisms of fresh oat harvested from Hongyuan on the Qinghai-Tibetan Plateau, where the altitude is 3,500 m a.s.l. Keshri et al. (2019) reported that *Pantoea* (34.7%) was the dominant genera in freshly chopped wheat plants, the RA of *Pantoea* increased to 46.3% after 6 h of ensiling, but then decreased continuously during silage maturation and they represented only 0.02% of the overall population at the terminal stage of 90 days. After 90 days of ensiling, *Pantoea* in oat silages from BM, BY, and DZ decreased to the second dominant bacterial genus. *Pantoea* species are gram-negative bacteria from the Enterobacteriaceae family, generally associated with plants, and are recognized as undesirable bacteria of silages because they can compete for nutrients with LAB (Li et al., 2018). In the study, *Pantoea* were not suppressed and remained at the high RA in BM, BY, DZ, and BR, however, it decreased to 1.4 and 0.4% in SC and SN, respectively. This was related to the flourishing of *Lactobacillus*, which confirmed the previous assumption that high-efficient LAB might be present in fresh oat sampled from high altitudes.

*Lactobacillus* was the minor genus in all fresh oat before ensiling, but its RA increased to 71.2 and 83.6% in oat silages of SC and SN, respectively. This was in line with the high acid concentration and low pH in oat silages of SC and SN. Previous studies also reported that *Lactobacillus* dominated the terminal silages (Guan et al., 2018; Hisham et al., 2022).

*Serratia* was the prevalent bacterial genus while *Kosakonia* was the minor bacterial genus in fresh oat, however, their RA increased during 90 days of ensiling. Little is known about the role of *Serratia* during ensiling, Li H. Z. et al. (2022) reported that *Serratia* could grow and survive under anaerobic conditions, and observed the increment of *Serratia* after ensiling. Duan et al. (2020) reported that *Serratia* spoiled chicken breasts were effectively inhibited by an antimicrobial substance produced by *Lactobacillus*. In the present study, the lower RA of *Serratis* in silage of SC and SN might be related to the dominant bacteria of *Lactobacillus*. Li H. Z. et al. (2022) also reported that silages with a higher RA of *Lactobacillus* showed a lower RA of *Serratia* and they proclaimed that *Serratia* was acid intolerant. *Kosakonia* is a genus of the Enterobacteriaceae family and has been observed by many researchers (Xiong et al., 2022). *Kosakonia* has been proven to have the ability to reduce ammonia nitrogen and volatile chemicals in silage (Zhang et al., 2021). In the present study, the lower ammonia N in oat silages of BY, DZ, and BR might be attributed to the higher RA of *Kosakonia* than other silages.

*Erwinia* was present in all fresh oat, however, certain species of *Erwinia* do not grow below pH 5.0 (Ogunade et al., 2018), supporting the decline of *Erwinia* after 90 days of ensiling in the present study. Enterobacteria, including *Erwinia herbicola* and *Rahnella aquitilis*, often were observed as dominant bacteria in fresh crops, however, they would be superseded by other genera during ensiling such as *Escherichia coli*, *Hafnia alvei*, and *Serratia fonticola* (Driehuis and Elferink, 2000).

*Paenibacillus*, *Pseudomonas*, and *Exiguobacterium* were detectable in all fresh oat, however, they disappeared in all silages. The genera of *Paenibacillus, Pseudomonas,* and *Exiguobacterium* were found in cold habitats (Rawat et al., 2020), they usually were inhibited during ensiling because of their intolerance to low pH (Borreani et al., 2013). Keshri et al. (2019) reported that *Pantoea*, *Weissella*, *Pseudomonas*, *Exiguobacterium*, and *Paenibacillus* dominated the bacterial community of fresh whole-crop wheat, however, they were replaced by *Lactobacillus* in the terminal silage.

*Leuconostoc* was the most dominant genus in fresh oat of BR, however, it was undetectable in fresh oat of other elevations. Cai et al. (1998) reported *Leuconostocs* were the most numerous and widely distributed on forage crops and silage. *Leuconostoc* could grow vigorously during the early stage of ensiling to initiate the silage fermentation, creating an aerobic and acidic environment for the proliferation of *Lactobacilli*. In the present study, it is not clear why *Leuconostoc* was the most dominant genus in fresh oat from BR rather than other elevations.

The study revealed the change in the fungal community between fresh and ensiled oat, the RA of *Dioszegia* in fresh oat increased with the elevation gradient and peaked at fresh oat from SC. This might be attributed to their cold-adapted properties. Trochine et al. (2017) isolated several species of the *Dioszegia* genus from glacier surface snow and found these yeasts showed strong cold-adapted capacity. After 90 days of ensiling, the RA of *Dioszegia* decreased, indicating they are unadaptable to the ensiling environment. Duniere et al. (2017) reported that the potential mycotoxigenic fungi of *Cladosporium* were the core microbiome in fresh small grains, but their RA markedly declined during ensiling. In the present study, the RA of *Cladosporium* spp. decreased during 90 days of ensiling. *Vishniacozyma* (also known as *Cryptococcus*) has been isolated from soil and wheat, but there are limited reports regarding this genus in silage. It is reported that *Vishniacozyma* could assimilate lactic acid and D-lactose (Tian et al., 2022; Li X. et al., 2022). *Vishniacozyma* was observed in both fresh and ensiled oat in the study, indicating the fresh oat might be subjected to soil contamination before ensiling. *Filobasidium* was detected in all fresh oat with the highest RA in fresh oat of DZ and BR, however, it markedly decreased after 90 days of ensiling. This is in agreement with the reports of Xu et al. (2019), who found that *Filobasidium* was an abundant genus in fresh corn and declined with the ensiling progress. Because of its psychrophilic abilities, *Cystofilobasidium* was detected in fresh oat harvested from the highest elevations with the lowest average annual temperature (Nakagawa et al., 2005). *Wickerhamomyces*, *Candida*, *Saccharomyces*, and *Rhodotorula* were the minor genus in fresh oat before ensiling, however, they became the major fungal community after 90 days of ensiling. Yang X. et al., (2022) studied the phyllosphere microbial communities and silage fermentation of *K. pygmaea* on the Tibetan Plateau and found that the fungal composition markedly changed: *Schizophyllum*, *Phodotorula*, *Rhodotorula*, *Candida*, and *Issatchenkia* became the most dominant fungal genera after 60 days of ensiling. Duniere et al. (2017) reported that members of the Saccharomycetales including *Candida*, *Wickerhamomyce*s, or *Saccharomyces* were the predominant fungi in terminal silage. *Wickerhamomyce*s are present in diverse habitats and frequently associated with the processing of food and grain products. Agarussi et al. (2022) reported that the predominance of fungi of *Wickerhamomyce*s during the fermentation of sorghum grains was due to their tolerance to extreme environmental conditions. *Saccharomycetales* spp. dominated the fungal community after 90 days of ensiling of small grain (Duniere et al., 2017). Yang X. et al. (2022) also reported that *Rhodotorula* became the dominant fungal genus in *K. pygmaea* 60-days silages harvested from 5,000 m a. s. l. This might be related to their cold-adaptability, Hu et al. (2015) isolated cold-tolerant yeast of *Rhodotorula mucilaginosa* from soil samples collected on the Qinghai-Tibet Plateau.

## Conclusion

The highest crude protein (CP) and lowest water-soluble carbohydrate (WSC) were observed in fresh oat from SN and BM, respectively, however, no distinct gradient trend in WSC and CP concentrations along the elevation gradient. The bimodal distributions of fungal and bacterial richness in fresh oat along the elevation gradient were observed, while the elevation gradients did not affect the fungal Shannon index in fresh oat. Oat from SN exhibited the best fermentation quality although the fresh oat of SN hosted the lowest LAB counts, indicating that high-efficient LAB might be present in fresh oat sampled from high altitudes. The high-efficient LAB can be further isolated from the Qinghai-Tibet Plateau, especially the higher elevational regions where the microorganisms hold a strong tolerance to the extreme environment.

## Data availability statement

The raw data supporting the conclusions of this article will be made available by the authors, without undue reservation.

## Author contributions

TS: conceiving the idea, designing the experiment, and writing, reviewing, and editing. YB and ZYa: sampling and analysis, data analysis, writing, reviewing, and editing. ZYu: formal analysis, methodology, review, and editing. RS and PX: writing, reviewing, and editing. ZYu, RS, and KF: sampling, analysis, and editing. All co-authors participated in discussions and revised the manuscript, contributed to the article, and approved the submitted version.

## Funding

This research was supported by the National Natural Science Foundation of China (31960355 and 32160780), The Tibetan Key R&D Program (XZ202001ZY0047N), and the State Key Laboratory of Barley and Yak Germplasm Resources and Genetics Improvement (Tibet Academy of Agricultural and Animal Husbandry Sciences) (XZNKY-2019-C-007K09).

## Conflict of interest

The authors declare that the research was conducted in the absence of any commercial or financial relationships that could be construed as a potential conflict of interest.

## Publisher’s note

All claims expressed in this article are solely those of the authors and do not necessarily represent those of their affiliated organizations, or those of the publisher, the editors and the reviewers. Any product that may be evaluated in this article, or claim that may be made by its manufacturer, is not guaranteed or endorsed by the publisher.

## Footnotes

^1^ http://drive5.com/uparse/
