# Supplementary material for: The microbial communities and natural fermentation quality of ensiling oat (Avena sativa L.) harvest from different elevations on the Qinghai-Tibet Plateau
Source: Front Microbiol. 2023 Jan 11;13:1108890. doi: 10.3389/fmicb.2022.1108890 (PMC9873999; doi:10.3389/fmicb.2022.1108890)
Supplement: Supplementary file 1 [file Table_1.docx]

Table S1. Climatic characteristics of sampling sites of oat (*Avena sativa* L.) on the Qinghai-Tibet Plateau

| Sampling location | Altitude(m) | Longitude | Latitude | Mean annual  temperature (℃) | Mean annual precipitation(mm) | Relative humidity  (%) |
| --- | --- | --- | --- | --- | --- | --- |
| Bomi County | 2852 | 95°29′49″ E | 30°60′46″ N | 11.3 | 51.2 | 67.9 |
| Bayi County | 3048 | 94°20′21″ E | 29°40′27″ N | 10.5 | 51.5 | 62.0 |
| Dazi County | 3737 | 91°36′25″ E | 29°48′33″ N | 8.48 | 63.6 | 45.0 |
| Biru County | 4022 | 93°38′37″ E | 31°45′48″ N | 10.7 | 50.5 | 66.8 |
| Suo County | 4100 | 92°20′37″ E | 31°41′17″ N | 10.7 | 50.5 | 66.8 |
| Seni County | 4447 | 92°10′13″ E | 31°26′30″ N | 10.5 | 51.6 | 62.0 |

Table S2. Alpha diversity of fungal and bacterial community diversities of fresh and ensiled Oat along the elevation gradient on the Tibetan Plateau.

| Item |  |  | Altitude^1^ | | | | | | SEM^2^ | *P*-value | Contrast *P*-values | |
| --- | --- | --- | --- | --- | --- | --- | --- | --- | --- | --- | --- | --- |
|  |  |  | BM | BY | DZ | BR | SC | SN |  |  | Linear | Quadratic |
| Fungi | Fresh | Shannon | 2.49 | 2.56 | 2.94 | 2.47 | 2.53 | 2.48 | 0.072 | 0.443 | 0.697 | 0.269 |
|  |  | Chao1 | 241 | 358 | 183 | 50.7 | 225 | 161 | 23.14 | <0.0001 | <0.0001 | 0.008 |
|  | Silage | Shannon | 1.17 | 2.68 | 3.04 | 2.91 | 0.41 | 3.27 | 0.268 | <0.0001 | 0.064 | 0.029 |
|  |  | Chao1 | 200 | 201 | 150 | 180 | 142 | 200 | 8.4 | 0.095 | 0.342 | 0.051 |
| Bacteria | Fresh | Shannon | 2.23 | 2.50 | 1.94 | 1.84 | 2.72 | 2.19 | 0.094 | 0.030 | 0.825 | 0.297 |
|  |  | Chao1 | 280 | 347 | 250 | 135 | 306 | 332 | 18.0 | <0.0001 | 0.896 | 0.0001 |
|  | Silage | Shannon | 1.76 | 2.52 | 2.10 | 1.57 | 1.84 | 1.09 | 0.137 | 0.030 | 0.016 | 0.060 |
|  |  | Chao1 | 239 | 285 | 193 | 166 | 266 | 238 | 15.6 | 0.234 | 0.770 | 0.241 |

^1^ BM, Bomi County; BY, Bayi County; DZ, Dazi County; BR, Biru County; SC, Suo County; SN, Seni County

^2^ SEM, standard error of the mean
